# Establishing Novel Antiretroviral Imaging for Hair to Elucidate Nonadherence: Protocol for a Single-Arm Cross-sectional Study

**DOI:** 10.2196/41188

**Published:** 2023-04-21

**Authors:** Amanda Poliseno, Ella Ferguson, Rose Perry, Alexandra Munson, Alexandra Davis, Lauren Hill, Jessica Keys, Nicole White, Claire Farel, Cynthia Gay, Carol Golin, Elias Rosen, Angela Kashuba

**Affiliations:** 1 University of North Carolina at Chapel Hill Chapel Hill, NC United States; 2 Division of General Internal Medicine School of Medicine University of North Carolina at Chapel Hill Chapel Hill, NC United States; 3 Department of Health Behavior Gillings School of Global Public Health University of North Carolina at Chapel Hill Chapel Hill, NC United States; 4 UNC Center for AIDS Research University of North Carolina at Chapel Hill Chapel Hill, NC United States; 5 Cecil G Sheps Center for Health Services Research University of North Carolina at Chapel Hill Chapel Hill, NC United States

**Keywords:** HIV, AIDS, adherence, hair, infrared matrix–assisted laser desorption electrospray ionization, IR-MALDESI, antiretroviral therapy

## Abstract

**Background:**

Adherence to antiretroviral (ARV) therapy is critical for achieving HIV RNA suppression in people living with HIV and for preventing HIV infection in uninfected individuals using preexposure prophylaxis. However, a high level of adherence can be challenging to achieve for people living with HIV on lifelong ARVs and for HIV-negative individuals using daily preexposure prophylaxis who are not at daily risk for HIV infection. Current biological measures of adherence are invasive and use bioanalytical methods that do not allow for real-time feedback during a clinic visit. This study was designed to test the feasibility and acceptability of using MedViewer, a novel, minimally invasive, hair-based assay that measures longitudinal ARV drug adherence in real time and provides an output for provider-patient discussion.

**Objective:**

The primary objectives were to investigate the feasibility of delivering the MedViewer results as planned, the acceptability of participation in a discussion of the MedViewer results, and the appropriateness of using MedViewer for adherence counseling. The secondary objectives were to investigate additional dimensions of feasibility, acceptability, and appropriateness of using the MedViewer test during a routine clinic visit for people with HIV.

**Methods:**

The proposed study was a single-arm cross-sectional study among patients receiving HIV care and providers of HIV care in a southeastern infectious disease clinic. The study originally planned to implement the MedViewer test with 50 eligible patients who were living with HIV across 2 viral load strata (undetectable or detectable plasma HIV RNA over the previous 2 years), administer brief visit-specific questionnaires to all patient and provider participants, and conduct qualitative in-depth interviews and quantitative end-line questionnaires with a subsample of patient participants (n=30) and all provider participants.

**Results:**

The Establishing Novel Antiretroviral Imaging for Hair to Elucidate Nonadherence study was funded by the National Institute of Allergy and Infectious Diseases and approved by the local institutional review board on November 4, 2019. Provider participant enrollment began on January 17, 2020, and patient participant enrollment began on January 22, 2020. Participant enrollment was halted on March 16, 2020, because of the COVID-19 pandemic (16 providers and 10 patients on study). Study activities resumed on February 2, 2021, with COVID-19 modifications approved by the local institutional review board. Participant enrollment closed on October 8, 2021, and data collection closed on November 15, 2021. In total, 36 unique patient participants, representing 37 samples, and 20 provider participants were enrolled. Data analysis and manuscript writing will take place throughout 2023.

**Conclusions:**

We anticipate that the data collected through this study will provide important insights regarding the feasibility, acceptability, and appropriateness of incorporating new real-time longitudinal, minimally invasive adherence tests into routine clinical care and identify potential barriers to medication adherence among patients.

**Trial Registration:**

ClinicalTrials.gov NCT04232540; https://clinicaltrials.gov/ct2/show/NCT04232540

**International Registered Report Identifier (IRRID):**

RR1-10.2196/41188

## Introduction

### Background

HIV RNA suppression in blood plasma is a key factor in controlling the HIV epidemic. Adherence to antiretroviral (ARV) therapy (ART) is an effective way to achieve viral suppression, maintain good health for people living with HIV, and prevent the transmission of HIV to uninfected individuals [1]. However, it can be challenging to maintain adherence to lifelong drug therapy. Poor adherence to ARVs can result in the development of viral resistance and worsened patient morbidity and mortality [2]. Having an accurate measure of ARV adherence for patients and providers can facilitate early interventions to improve adherence. Blood plasma or intracellular ARV concentration monitoring has been considered the “gold standard” for biological measures of adherence. However, this approach has its own set of limitations, including being invasive, requiring advanced processing (eg, intracellular measures) or specific storage conditions, being a short-term measure of drug-taking behavior (depending on the half-life of the analyte), and requiring long turnaround times [3]. Therefore, valid noninvasive longitudinal measures of adherence such as hair analysis are critical for optimizing ARV effectiveness.

In this study, we proposed a novel solution to bridge the gap in adherence monitoring to improve clinical outcomes based on hair. Hair is unique in that it has the potential to provide information about drug intake over a longer period compared with other biological fluids, including plasma [4], blood cells [5], and urine [6]. Recent studies have used sensitive analysis of hair strands through liquid chromatography–mass spectrometry to evaluate ARV concentrations. These studies have demonstrated that ARV drug concentrations scale proportionally with dose frequency [7] and can predict virologic success [8]. Liquid chromatography–mass spectrometry methods typically evaluate hair segments of ≥1 cm; this length corresponds to at least a month of hair growth. Our approach uses infrared (IR) matrix–assisted laser desorption electrospray ionization (MALDESI) technology for mass spectrometry imaging (MSI) to visualize and quantify ARV concentrations longitudinally over the previous month of hair growth. An IR-MALDESI MSI method has been validated for the quantification of emtricitabine (FTC) and dolutegravir (DTG) in hair strands. As part of this validation, we have determined the lower limits of quantification for each of these 2 ARVs (FTC: 0.27 ng/mg; DTG: 0.04 ng/mg) using a series of prepared hair standards and ensured that our sensitivity is within the range of incurred samples we have evaluated as part of study aim 1. The benchmarking of this IR-MALDESI MSI longitudinal ARV profiling has been conducted through a 3-phase (single dose, daily dose, and dose proportionality) directly observed therapy study with FTC+tenofovir and DTG (ClinicalTrials.gov NCT03218592). Our preliminary results suggest that this method has potential value in measuring longitudinal drug exposure [9] and distinguishing between different adherence patterns [10].

### Objectives

For ease of communicating regarding the IR-MALDESI MSI approach with patients and providers, we named this approach “MedViewer” and will refer to it as such throughout this protocol paper. In preliminary studies, we demonstrated that MedViewer can rapidly and accurately provide noninvasive and longitudinal evidence of drug ingestion and thereby has the potential to provide clinicians, researchers, patients, and study participants with feedback on adherence performance [3]. However, little is known about how such a tool would be accepted as an adherence-enhancing intervention by patients or providers or how best to implement it feasibly in the context of a clinic setting. The primary objectives of this study were to investigate the feasibility of delivering the MedViewer analysis as planned, the acceptability of discussing the MedViewer results, and the appropriateness of MedViewer use for adherence counseling. Secondary objectives of this study were to investigate additional dimensions of feasibility, acceptability, and appropriateness of using MedViewer to provide feedback to people living with HIV regarding longitudinal patterns of medication adherence. The goal of this work was to develop a simple, rapid, noninvasive, longitudinal monitoring tool of ARV adherence that will provide useful feedback for clinicians and patients to improve ARV adherence. As this was a feasibility study, of note, no change in clinical care was initiated based on information assessed with the intervention.

## Methods

### Intervention

The study intervention included four components: (1) a standardized training session for medical providers; (2) an informational video for patients; (3) the hair sample, MedViewer test, and accompanying MedViewer report (patient and provider versions); and (4) communication aids for providers.

All research activities were conducted in as private a setting as possible.

All medical providers that consented to participate in this study attended a standardized training session. Training sessions were offered in person and in a web-based format. The training sessions lasted between 30 and 60 minutes and were held at the start of the study as well as at various points throughout the study (for providers who were new or had not enrolled at the study start). The training introduced the providers to the MedViewer patient video (described in the following paragraph), the MedViewer test and report, and provider communication aids. The training also prepared providers to incorporate the delivery of the investigational MedViewer test results into discussions with patients during routine HIV care appointments by providing an opportunity to practice interpreting and discussing MedViewer results with patients using an example MedViewer report. The training session was required for all enrolled providers participating in the study before they could be scheduled to see enrolled patients for visits to review their MedViewer report. Supplemental training sessions with the same training materials were offered as needed. Although providers used the investigational MedViewer reports during their conversations with patients about ARV adherence, provider participants still only used currently accepted measures of ARV adherence (eg, plasma HIV RNA, HIV genotyping, and plasma therapeutic drug monitoring of ARVs) for making clinical decisions.

An 8-minute informational video was used to introduce patients to the MedViewer test, the hair collection process, and how test results can be useful for conversations between patients and providers about adherence. More specifically, the video explained (1) how ARVs are processed in the body and end up in the hair, (2) what the hair sample collection process would be like, (3) how the hair sample would be processed and how data and reports would be generated, (4) how the hair sample would be disposed of, (5) how the provider might review the results with the patient, and (6) how the test results could inform conversations about the patient’s adherence. The video also addressed potential patient concerns about the test (as identified in a formative study conducted as part of intervention development) [11]. Potential concerns included but were not limited to whether it was painful to provide a hair sample and how patient privacy would be protected. This video was part of the informed consent process for patient participants to facilitate their understanding of the MedViewer aspect of the study. Eligible and interested patients watched the video during the informed consent process either in a private room in the research center or clinic or via an institutional review board (IRB)–approved videoconferencing call. Patients had the opportunity to watch the video multiple times as needed.

After providing informed consent, patient participants provided a hair sample for MedViewer testing. Hair sample collection took place in the research center or clinic initially. After the pandemic, hair sample collection could also take place in a private remote location where the patient participant felt comfortable, such as their home, to minimize study participants’ exposure to the clinic environment. To collect the sample, a trained clinical research staff member used tweezers to pluck 5 strands of hair from the back of the patient’s head. The research team member then placed the hair on a foil package and affixed the distal end of the hair with a label. The folded foil was placed into a resealable biohazard bag in a closed container and then transported promptly to the laboratory for testing in accordance with local environmental, health, and safety policies. The date and time of sample collection and transportation were documented on a paper case report form. At the laboratory, the laboratory scientist placed the sample in the IR-MALDESI instrument to run the test [3].

**Figure 1:**
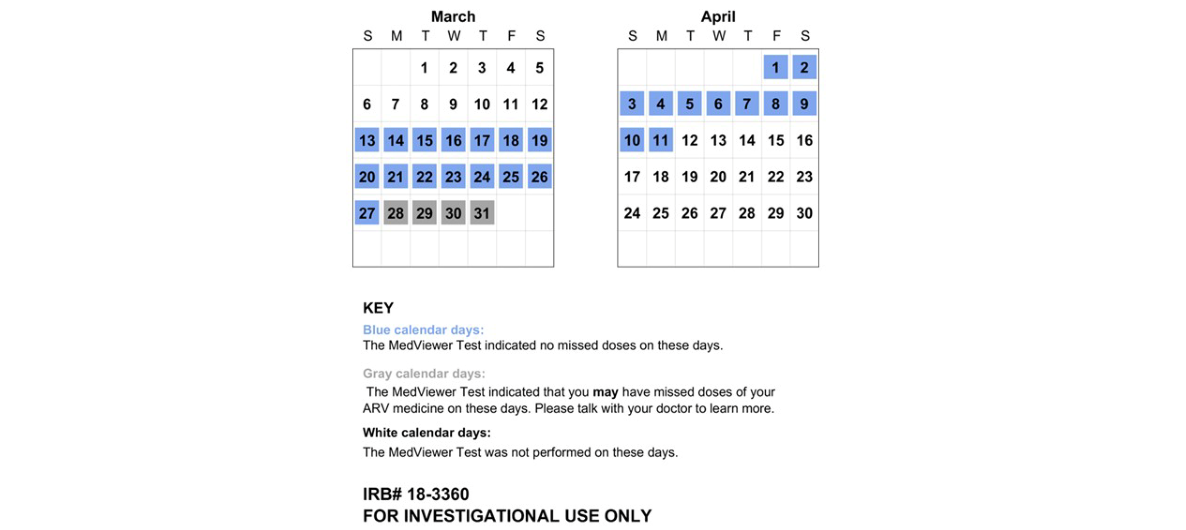
Example patient MedViewer report. ARV: antiretroviral; IRB: institutional review board.

**Figure 2:**
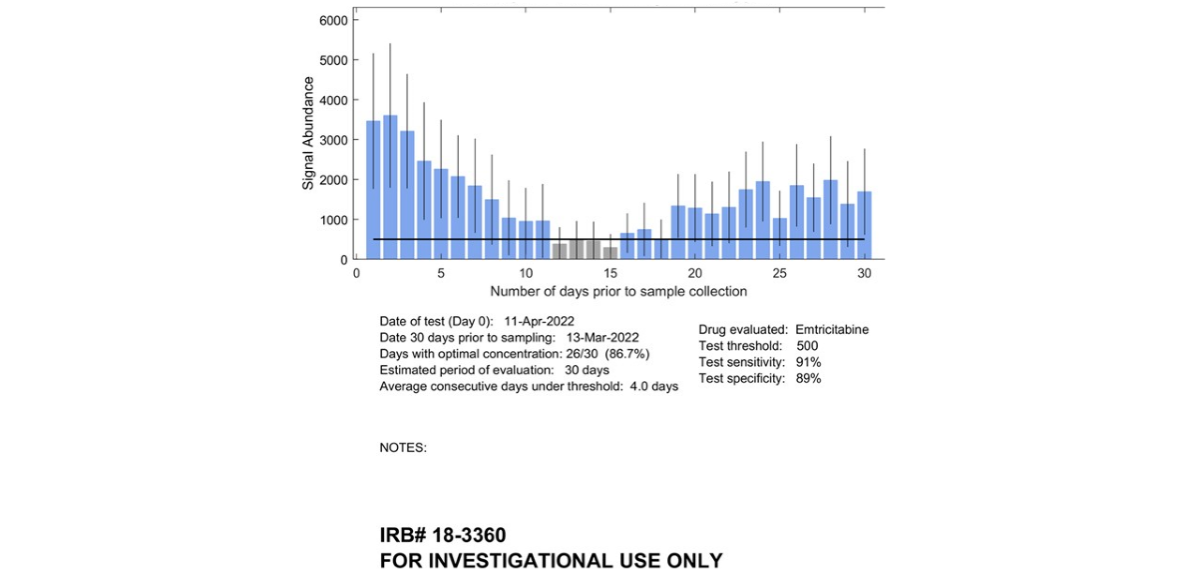
Example provider MedViewer report. IRB: institutional review board.

As suggested by our formative research findings [11,12], after the hair sample was run in the IR-MALDESI instrument, the laboratory scientist generated 2 distinct visual reports of the results intended for patients (Figure 1) and providers (Figure 2). The patient version presented a calendar indicating whether the IR-MALDESI output from each day achieved a sufficient threshold consistent with daily dosing, whereas the provider version displayed more detailed quantification of the IR-MALDESI output with a bar chart. The laboratory scientist then sent both reports via secure email to the research team, who printed the reports and delivered a hard copy or electronic version to the appropriate provider participant in the infectious disease (ID) clinic. The laboratory scientist that ran the test recorded the time the analysis process was started and the time the report was available for the research team on the same paper case report form. These times were used to assess the duration of this component of the intervention.

Before seeing the patient, the provider reviewed the provider and patient reports. During the patient’s regularly scheduled appointment at the ID clinic, the provider and patient viewed the patient version of the report together and used them to have a conversation about the patient’s ARV medication adherence. If the provider thought that the patient would be interested in the more detailed provider report, the provider could elect to also review that report with the patient. If the MedViewer report was not available during the patient’s regularly scheduled ID clinic appointment, the provider and patient participants had the option to discuss them during a separately scheduled MedViewer appointment within 4 weeks of hair sample collection. The provider participants did not follow a standardized script to discuss the results with the patient; rather, the provider conducted the appointment based on their clinical judgment and discretion, drawing on information from the provider training session and communication aids, medical expertise, and their clinical judgment regarding the individual needs and circumstances of the patient participant (Figure S1 in Multimedia Appendix 1). If the provider chose to follow suggested communication strategies listed in the MedViewer intervention reference sheet, they could discuss MedViewer reports with patients by explaining the summary statistics printed with the results or pointing out any patterns of insufficient drug concentrations and adherence successes, asking patients about event-level or chronic and psychosocial causes of missed doses, asking patients about successful adherence strategies, working with patients to identify personalized strategies that could help them overcome causes of missed doses in the future or to replicate successes, offering encouragement for good adherence, and working with patients to set goals for improving future adherence.

Owing to their investigational nature, the MedViewer reports were not entered into the patients’ electronic health records, did not become a formal part of the clinical patient record, and were not used for clinical decision-making. After the provider and patient reviewed the MedViewer reports, both versions of the report were destroyed in accordance with standard medical document destruction procedures. A copy of each was stored in the research record. The research team documented receipt of the MedViewer report by the provider on the study forms.

### Recruitment

#### Recruitment and Retention Strategies for Patient Participants

We planned to enroll 50 patient participants from a large hospital-based outpatient ID clinic in the southeastern United States. Data collection occurred in the clinic, in a clinical research center, or at remote locations to minimize exposure to the clinic environment in response to the COVID-19 pandemic. The ID clinic has a full-time research screener to assess patient eligibility for open research projects. Of all the patients in the ID clinic who are living with HIV, approximately 95% have consented to having their patient information available in a secure clinic database from which it can be viewed by a research screener to identify potential eligibility for open research studies and to being notified of studies for which they are potentially eligible (as per IRB form 99-MED-408). Patients in the database have indicated all the methods of contact to which they have and have not agreed for contact by researchers for potential participation. We used an IRB-approved screening process that has been used in the ID clinic for >10 years whereby the clinic screener or a trained research team member prescreened patients scheduled for an appointment in the upcoming week in the clinic database. After using the clinic database to identify potential patients, the screener or trained research team member assessed the patients’ electronic health record for eligibility criteria under an IRB-approved limited waiver of the Health Insurance Portability and Accountability Act (HIPAA). At regular intervals, the clinic screener shared with the research team the list of scheduled patients who met eligibility at prescreening.

Using the list of potentially eligible study participants, the research team contacted patients by phone before their next scheduled HIV appointment using an IRB-approved phone screening questionnaire to notify them about the study and assess their interest. Phone calls were conducted in a private room to prevent inadvertent disclosure of participant information. Participants could also contact study staff in response to IRB-approved study recruitment flyers. For participants who expressed interest on the phone, the researchers continued with the phone contact to verify their eligibility using a brief standardized IRB-approved script and screening form (with questions pertaining to patient age, amount of time as a patient at the University of North Carolina at Chapel Hill [UNC] ID clinic, HIV appointments in the last year, current and past prescribed ARV medications, and length of caput hair), and if confirmed to be eligible for a screening visit and the patient agreed, they provided basic demographic data needed to schedule their appointment in the clinical research management system. This was done under an IRB-approved limited HIPAA waiver. Full HIPAA and informed consent occurred during the first patient participant visit. All contact attempts were documented in a recruitment log.

To ensure a sufficient number of patients with a history of detectable viral loads (VLs; who represented a minority in the clinic), we enrolled participants into 2 VL groups with a target of 25 participants in each group. Group A included those with plasma HIV RNA below the limit of quantification (<40 copies per million) over the previous 2 years with documentation of at least one test in the previous 6 months. Group B included those who had had at least one plasma HIV RNA result above the limit of quantification within the previous 2 years. We anticipated enrolling an average of 7 patients per month over the course of 7.5 months (33 weeks) to reach our target of 50 total participants. We screened and enrolled patients of all gender identities, racial identities, ethnicities, and ages (>18 years) such that the demographic distribution of screened participants reflected that of patients at the UNC ID clinic who are living with HIV.

#### Recruitment and Retention Procedures for the In-depth Interview Subsample of up to 30 Patient Participants

We also planned to enroll a subsample of up to 30 patient participants (up to 15 per VL group) enrolled in the larger study, to participate in a follow-up study visit including an in-depth interview (IDI) and brief end-line questionnaire. We invited every enrolled patient to participate.

#### Recruitment and Retention Procedures for Provider Participants

We aimed to recruit and enroll all (up to 30) medical providers who provided care to patients in the ID clinic (eg, attending physicians, fellows, nurse practitioners, physician assistants, nurses, and pharmacists) during the study period.

Most of our initial provider participant contacts were completed through email, wherein an IRB-approved email invitation to attend the provider training and participate in the study was sent. If the provider was willing to participate, they scheduled their training with the research team and completed the informed consent process before the training. Full informed consent and screening occurred before the provider training.

### Ethics Approval

This study received ethical approval from the University of North Carolina at Chapel Hill instituional review board (reference ID 336881).

### Informed Consent, Compensation, and Confidentiality

The investigators obtained informed consent from each patient and provider participant before starting any study procedures according to the standards set forth in the International Conference on Harmonization Good Clinical Practice guidelines and per local standard operating procedures. The process included reviewing consent forms with potential participants in a confidential setting and explaining all risks and benefits associated with participation in the study. The IRB-approved consent form was read with the participant in a private space, after which questions were solicited from the participant. The participant was allowed time alone to rereview the form and questions were again solicited. To ensure understanding, study staff asked questions of the participants regarding study procedures. The consent forms used language sufficiently simple for lay persons to comprehend. Participants were not coerced into taking part. Children aged <18 years, adults with impaired decision-making skills, and non-English speakers were not enrolled in this study. Each participant was provided with a photocopy of all the documents they signed. The informed consent process covered all elements required by research regulations. In addition, the process specifically addressed the following topics of importance to this study: (1) the unknown safety and unproven efficacy of the study interventions, (2) the importance of patients in both study groups to the success of the study, (3) the importance of adherence to the study visit and procedure schedule, (4) the potential medical risks of study participation (and what to do if such risks were experienced), (5) the potential social harms associated with study participation (and what to do if such harms were experienced), (6) the limited benefits of study participation, (7) the distinction between research and clinical care, and (8) the right to withdraw from the study at any time.

During the consent process, the patient participants watched the aforementioned short, IRB-approved video describing MedViewer. Participants who were not able to demonstrate adequate understanding of key concepts after exhaustive educational efforts were not enrolled in the study. The informed consent process included an assessment, through a series of questions, of each potential participant’s understanding before enrollment and sequential assignment of concepts identified by the protocol team as essential to the informed consent decision.

Finally, the participants were offered the opportunity to sign the consent form or provide verbal consent for follow-up IDI study visits using an IRB-approved verbal consent process. All participants also reviewed and signed a HIPAA form approved by the IRB.

Participants were compensated for all parts of the study that they completed. Payment was provided in the form of Visa gift cards, and the amount per activity was reviewed and approved by the local IRB to not be coercive. Patient participants received US $5.00 for providing a hair sample, US $15.00 for providing a blood sample, US $20.00 for completing the baseline questionnaire, and US $10.00 for completing the postvisit questionnaire. When study operations were resumed after the COVID-19 pandemic, patient participants were provided with an additional US $20.00 to cover any telephone minutes or internet data they may have had to purchase to complete study activities. If a patient participant did not complete all parts of the study, their payment was adjusted. If the patient participant agreed to participate in the subsample, they were compensated for these activities as well. Patient participants received US $30.00 for taking part in the IDIs and US $10.00 for the questionnaire administered at the end of the interview. As these interviews were conducted in a web-based format after the COVID-19 pandemic, an additional US $20.00 was added to the gift cards to cover any telephone minutes or internet data they may have had to purchase to complete study activities. Provider participants received US $20.00 for attending the provider training session and completing all baseline questionnaires, US $30.00 for completing the postvisit questionnaires for all MedViewer patient visits they conducted during the study, US $20.00 for completing the IDI, and US $10.00 for completing the end-line questionnaire.

Confidentiality was maintained throughout the study by storing all specimens for current and future use with a unique identifying number, which was linked to the participant’s name, social security number, address, telephone number, and hospital medical record number. The principal investigators and study staff were the only people with access to the identifying information. Any information provided to other people working on this study was given with the study ID number, not other identifying information. The records were secured in a locked file cabinet in a locked room in a badge access–only office suite of the principal investigator.

All electronic data for this study were stored on a dedicated university server with extensive protections and securities that exceed the standards of the UNC privacy of electronic information policy.

Participant confidentiality and privacy were strictly held in trust by the participating investigators, their staff, and the sponsors and their interventions. This confidentiality was extended to cover testing of biological samples in addition to the clinical information related to participants. Therefore, the study protocol, documentation, data, and all other information generated were held in strict confidence. No information concerning the study or the data was released to any unauthorized third party without previous written approval of the sponsor.

The study participants’ contact information was securely stored at each clinical site for internal use during the study. At the end of the study, all records continued to be kept in a secure location for as long a period (6 years after completion of the research) as dictated by the reviewing IRB, institutional policies, or sponsor requirements. The period of 6 years is consistent with the requirements of Title 45 of the Code of Federal Regulations 46.115(b) and Title 21 of the Code of Federal Regulations 56.115.

To further protect the privacy of study participants, a Certificate of Confidentiality was issued by the National Institutes of Health. This certificate protects identifiable research information from forced disclosure. It allows the investigator and others who have access to research records to refuse to disclose identifying information on research participation in any civil, criminal, administrative, legislative, or other proceeding whether at the federal, state, or local level. By protecting researchers and institutions from being compelled to disclose information that would identify research participants, Certificates of Confidentiality help achieve the research objectives and promote participation in studies by helping assure confidentiality and privacy to participants.

### Study Objectives and End Points

The study objectives and end points are outlined in Table 1.

**Table 1:** Objectives and end points for the study.

Objectives			End points		Justification for end points
**Primary**					
	Investigate the feasibility of delivering the MedViewer intervention as planned, the acceptability to patients of participation in the MedViewer intervention, and the appropriateness of MedViewer use for adherence counseling	Feasibility: proportion of participants receiving the MedViewer report during their provider visit as planned (ie, the results are delivered to the designated research staff member within 2 hours of initiation of hair processing and the results are discussed with the provider or pharmacist within 4 weeks of hair collection) Acceptability: proportion of contacted patients who are eligible for a screening visit (not including inclusion criterion 9) who agree to participate in the MedViewer intervention pilot study Appropriateness: perceived usefulness of the MedViewer intervention for adherence counseling		Evaluation of these implementation domains will indicate the potential for future application of MedViewer in routine care and help identify needed modifications to the MedViewer intervention to improve delivery. The framework by Proctor et al [13] for outcomes in implementation research indicates the importance of assessing feasibility, acceptability, and appropriateness.	
**Secondary**					
	Investigate additional dimensions of feasibility, acceptability, and appropriateness of using hair IR^a^-MALDESI^b^ MSI^c^ (MedViewer) to provide patients living with HIV with feedback regarding longitudinal patterns of medication adherence	Feasibility: Reasons for patients’ nonreceipt of the MedViewer report during a visit with the provider or pharmacist within 4 weeks of hair collection (if applicable) Reasons for nondiscussion of the MedViewer report with the provider or pharmacist (if applicable) Length of time (in minutes) from initiation of hair processing to MedViewer report delivery to the designated research staff member Acceptability: Provider-reported likelihood of recommending MedViewer to future patients Patient-reported likelihood of agreeing to future MedViewer use Patient comprehension of the MedViewer report Appropriateness: Perceived usefulness of MedViewer to promote ART^d^ adherence Perceived impact of MedViewer use on patient-provider communication and relationship		Evaluation of these implementation domains will indicate the potential for future application of MedViewer in routine care and help identify needed modifications to the MedViewer procedure to improve delivery. The framework by Proctor et al [13] for outcomes in implementation research indicates the importance of assessing feasibility, acceptability, and appropriateness.	
**Exploratory**					
	Assess exploratory aspects of MedViewer feasibility and acceptability and patient and provider views of and experiences with the MedViewer test Explore the impact of adherence counseling using MedViewer on ART adherence and hypothesized mechanisms of change (adherence information, motivation, and behavioral skills)	Feasibility: Patient-reported maximum out-of-pocket cost willing to pay for MedViewer Cost of MedViewer delivery per person Compatibility of MedViewer with current clinic practices Acceptability: Patient-reported reasons for declining participation in the MedViewer intervention pilot study Reasons why participants would or would not agree to (patient-reported) or recommend (provider-reported) future MedViewer use Acceptability of specific components of the MedViewer procedure: Sufficiency of provider training and materials Sufficiency of patient education (video) Provider satisfaction with results delivery (discussion format and content) Patient satisfaction with results delivery (person, discussion format, and content) Adherence-related: ART adherence measured via self-reported assessment of 3-, 7-, and 30-day adherence Adherence information (qualitative assessment of information and understanding gained of patient adherence resulting from use of MedViewer) Adherence motivation (quantitative and qualitative assessment of adherence motivation resulting from use of MedViewer) Adherence behavioral skills (quantitative and qualitative assessment of adherence behavioral skills and self-efficacy resulting from use of MedViewer)		Preliminary assessment of MedViewer’s impact on the intended behavioral outcome (ART adherence) and hypothesized mechanisms of influence (based on the Information–Motivation–Behavioral Skills model) will be used to inform the design of a future randomized trial to evaluate the effect of MedViewer on these end points [14].	
	Further explore the accuracy of the MedViewer test as an adherence measure	ARV^e^ concentrations in blood collected for first patient study visit and follow-up patient study visit when applicable Patient viral load assessed with a clinical care visit linked to a patient-provider MedViewer visit and abstracted from the medical record by study staff log10 ARV drug response in hair via IR-MALDESI collected for first patient study visit and follow-up patient study visit when applicable		These end points will allow for comparison of reported concentrations of medication in hair with recent ART adherence, as measured via Mitra and current viral load. These comparisons will provide further evidence to assess MedViewer accuracy (in addition to that gathered in aims 1 and 2) among a larger group of patients.	

^a^IR: infrared.

^b^MALDESI: matrix-assisted laser desorption electrospray ionization.

^c^MSI: mass spectrometry imaging.

^d^ART: antiretroviral therapy.

^e^ARV: antiretroviral.

### Assessments

#### Data Collection Procedures

**Figure 3:**
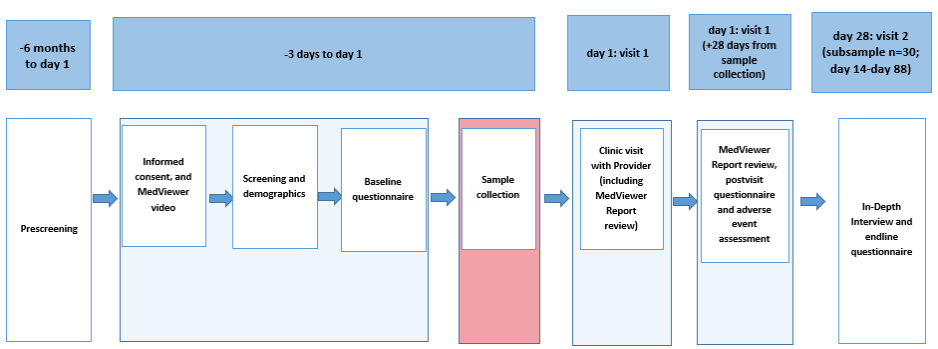
Patient participant study schema. Sequence of the Establishing Novel Antiretroviral Imaging for Hair to Elucidate Nonadherence study activities for patient participants. Note: MedViewer Report review can be conducted up to 28 days from hair sample collection if not completed at day 1 clinic visit with providers.

**Figure 4:**
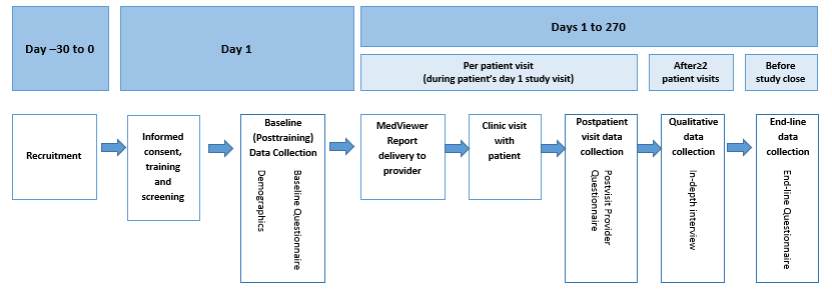
Provider participant study schema. Sequence of the Establishing Novel Antiretroviral Imaging for Hair to Elucidate Nonadherence study activities for provider participants.

Patient and provider participants were administered IRB-approved questionnaires at different points during their study participation to assess outcome measures (Figures 3 and 4). These questionnaires were designed by trained qualitative researchers. Patient participants were administered a baseline computer-assisted self-interviewing (CASI) questionnaire after providing informed consent but before receiving and discussing the MedViewer reports with their provider. This questionnaire contained items asking about self-rated health, patient sociodemographic information (time to travel from home to the ID clinic, age, sex assigned at birth and gender identity, sexual orientation, race and ethnicity, current marital status, education level, income in the previous year, current employment status, health insurance status and type, and method of paying for ARVs), comprehension and sufficiency of video content, self-reported ARV adherence over the previous 30 days, and adherence motivation and self-efficacy. After receiving and discussing the MedViewer report with their provider, patient participants were administered a postvisit CASI questionnaire. This 13-item questionnaire comprised questions regarding experience receiving and discussing the MedViewer report, with whom they had discussed the report, their comprehension of the report, and acceptability of the process.

The subsample (up to 15 from each VL group) of patient participants who returned for an IDI completed semistructured IDIs either in person or remotely via an IRB-approved videoconference. Interview topics included perceived usefulness of the MedViewer report for adherence counseling; reasons why the patient participant would or would not use MedViewer in the future if available; satisfaction with the patient education video; comprehension of the MedViewer report; satisfaction with adherence counseling discussion using the MedViewer report; attitude toward wait time and hair sample collection process; anticipated effect of regular MedViewer report use on patient-provider relationships and communication; and perceived effect of the MedViewer report on comprehension of own adherence behavior, adherence motivation, and adherence behavioral skills. All IDIs were approximately 1 hour in length. Trained research staff experienced in qualitative research conducted the IDIs using a semistructured interview guide that included open-ended questions corresponding to each qualitative outcome. Interviewers had the flexibility to probe patient responses and pursue discussion diverging from the initial interview questions if relevant to the outcomes of interest. Each interview was digitally recorded and transcribed verbatim for analysis. After completing the IDI, patient participants also completed a brief CASI questionnaire pertaining to their perceived adherence over the previous 3, 7, and 30 days as well as their adherence motivation and maximum out-of-pocket cost that they would be willing to pay for future MedViewer reports.

After completing the provider training session, provider participants were asked to complete a short baseline questionnaire. The questions pertained to their satisfaction with the training quality, content, perceived knowledge gained of the MedViewer test and study procedures, and self-efficacy to deliver the MedViewer report to patients. Each time a provider saw a patient participant for a MedViewer report discussion, they were asked to complete a postvisit questionnaire. These questionnaires assessed if the MedViewer reports were discussed, how long the discussion took, their perception of the patient’s comprehension of the MedViewer report, and their perceived usefulness of the MedViewer report as an adherence tool. All provider participants enrolled in the study were asked to complete an IDI with research staff either after having reviewed the report with at least 2 patients or before study close, depending on which occurred first. The topics of the provider IDIs included the usefulness of the MedViewer report in encouraging patients to sustain or improve adherence, reasons why they would or would not recommend future MedViewer use, their satisfaction with the MedViewer report and adherence counseling using the report, perceived ease of delivering and discussing MedViewer results during a typical appointment, perceived level of disruption to clinic flow by MedViewer procedures, and the anticipated effect of regular MedViewer use on patient-provider communication and relationships. All provider IDIs were audio recorded with participant consent, and the audio file was transcribed for analysis. At the end of a provider’s participation in the study, they were asked to complete an end-line questionnaire. The items on this questionnaire pertained to the likelihood of recommending MedViewer to other patients, satisfaction with adherence counseling discussions using the MedViewer report, perceptions of the most appropriate clinician for future MedViewer counseling, perceived usefulness of the MedViewer report in encouraging patients to sustain or improve adherence, and the influence of the MedViewer report on patient-provider communication and relationships.

#### Outcome Variables and Measures

##### Primary Outcomes

###### Feasibility

- Proportion of Patient Participants Receiving the MedViewer Report During Their Clinic Visit as Planned

This primary feasibility outcome is the proportion of participants who both (1) had their MedViewer report delivered to the designated research staff member within 2 hours of initiation of hair processing and (2) discussed the results with a provider (ie, their medical provider or the HIV care pharmacist) within 4 weeks of hair collection. To assess this primary feasibility outcome, the numerator was calculated using 2 separate data sources. First, to assess whether the designated research staff member received the patient’s MedViewer report within 2 hours of initiation of hair processing, a study tracking log was maintained by the study team on which the following times were recorded: hair collection, hair transport, hair processing, and delivery of the report to the designated research staff member. The patient participants were asked to indicate on the self-report postvisit questionnaires whether the MedViewer report was discussed following clinic visits with their medical provider or the HIV care pharmacist; the study team also noted which clinician reviewed the report with the patient participant. As a backup data source, providers were also asked in their postvisit questionnaire if they discussed the report with the patient. The numerator of the primary feasibility outcome was a single dichotomous (yes or no) measure. Patients were counted in the numerator of the primary outcome if (1) the study team member indicated on the tracking log that, yes, study staff received the patient’s results within 2 hours of initiation of hair processing in the laboratory—this was determined by subtracting time 1 (when hair processing was initiated) from time 2 (when the results were delivered to the designated research staff member—and (2) the patient self-reported on the postvisit questionnaire that, yes, they discussed the results with the provider. The proportion of enrolled participants who achieved the primary feasibility outcome will be estimated. An estimated proportion and corresponding 95% Wilson-Score CI was used to analyze this outcome and all similar dichotomous (ie, binary) outcomes.

###### Acceptability

The acceptability primary outcome was assessed based on the proportion of contacted patients who were eligible and agreed to participate in the MedViewer intervention feasibility study. Data for this outcome were obtained from the recruitment log. This was a single dichotomous (yes or no) measure for each individual participant. To create this measure, the denominator included all potential patient participants who were contacted and found to be eligible for the study as documented on their prescreening questionnaire. The numerator was those who agreed to participate in the study as documented by their informed consent form. The acceptability primary outcome is descriptive. The proportion of potential patient participants eligible for a screening visit who achieved the acceptability primary outcome (accepted enrollment) was estimated. An estimated proportion and corresponding 95% Wilson-Score CI was used to analyze this outcome.

###### Appropriateness

The appropriateness primary outcome (perceived usefulness of MedViewer for adherence counseling) was assessed using data obtained from the IDIs.

##### Secondary Outcomes

###### Feasibility

- Reasons for Patient’s Nonreceipt of the MedViewer Report During a Visit With Provider or Pharmacist Within 4 Weeks of Hair Collection

This secondary feasibility outcome was only assessed for the subsample of patient participants for whom nonreceipt of the MedViewer report was reported (either by a provider in the provider postvisit questionnaire or by a research team member in a study activity tracking log). Reasons for nonreceipt were assessed using a 1-item multiple-choice question with a “check all that apply” approach in the provider postvisit questionnaires and an open-ended response on study tracking forms completed by the research team.

- Length of Time (in Minutes) From Initiation of Hair Processing to MedViewer Report Delivery to Designated Research Staff Member

This secondary feasibility outcome was defined as the amount of time elapsed (in minutes) from the time of initiation of hair processing in the laboratory to the time the results were delivered to the designated research staff member. This variable was determined by subtracting time 1 (when hair processing was initiated) from time 2 (when the results were delivered to the designated research staff member). This variable was measured using the study visit hair sample tracking log. The log was maintained by the study team, who reported the time at which each study activity occurred.

###### Acceptability

- Provider-Reported Likelihood of Recommending MedViewer to Future Patients

This secondary acceptability outcome was assessed using a single Likert-type item among providers in a self-reported end-line questionnaire administered by study team members. Providers were asked to rate their likelihood of recommending MedViewer, if available for regular use, to other patients in the future. This item was rated on a 4-point scale (*definitely would not recommend* to *definitely would recommend*). We calculated the proportion of providers reporting each response category (count/denominator and percentage).

- Patient-Reported Likelihood of Agreeing to Future MedViewer Use

This secondary acceptability outcome was assessed among patients in the self-reported postvisit questionnaire administered by the study team members after the participant had received and discussed their MedViewer report with their provider (or completed their visit without a discussion). This was measured using a Likert-type item. Participants were asked to rate their future likelihood of agreeing to MedViewer use if recommended by a provider. This item was rated on a 4-point scale (*definitely would not use* to *definitely would use*). We calculated the proportion of patients reporting each response category (count/denominator and percentage).

- Patient Comprehension of MedViewer Report as Perceived by Patients and Providers

This secondary acceptability outcome was assessed in postvisit questionnaires and IDIs. In the postvisit questionnaire following the MedViewer report discussion, patients were asked to rate their comprehension of the information presented in the report. Providers completed a parallel item in their postvisit questionnaire to assess their view of how well patients understood the information in the MedViewer report. The patient item was rated on a 4-point scale (*very difficult to understand* to *very easy to understand*). The provider item was rated on a 5-point scale (*understood poorly* to *understood excellently*). We calculated the proportion of patients reporting each response category (count/denominator and percentage). We calculated similar proportions for provider responses regarding their perception of each patient’s comprehension as a proportion of patients. Patient comprehension of the report was also assessed qualitatively—during the patient IDIs, we conducted a cognitive interview to assess patient comprehension of adherence information presented in the MedViewer report. We used the data from the IDIs to summarize the extent to which patients understood the content of the reports and describe the primary content and formatting features of the report that were poorly understood by participants, if applicable.

###### Appropriateness

- Perceived Usefulness of MedViewer to Promote ART Adherence

This secondary appropriateness outcome was measured in the patient postvisit questionnaire administered by the study team members following the MedViewer discussion, in the provider postvisit questionnaire following each visit with an enrolled patient, in the patient and provider end-line questionnaires, and in the patient and provider IDIs. Participants were asked to rate their agreement with statements regarding MedViewer’s usefulness in promoting adherence over the following 30 days, adherence motivation, skills, and strategies to avoid missed doses. These items were rated on a 5-point scale (*not at all useful* to *extremely useful*). For each item, we calculated the proportion of patients and providers reporting each response category (count/denominator and percentage).

- Perceived Impact of MedViewer Use on Patient-Provider Communication and Relationship

This secondary appropriateness outcome was assessed using the postvisit questionnaires for patients and the end-line questionnaires for providers as well as through questions during the IDIs. In the postvisit questionnaires administered to patients following the MedViewer visit, patients were asked to rate the comparative satisfaction with the patient-provider interaction during the MedViewer visit as compared with a typical visit. In the end-line questionnaires administered to providers, they were asked to rate the effect of using MedViewer on their relationships with their patients. The patient questionnaire item was rated on a 5-point scale (*much less satisfied than usual* to *much more satisfied than usual*), and the provider questionnaire item was rated on a 5-point scale (*very negatively affected relationships* to *very positively affected relationships*). For each questionnaire item, we calculated the proportion of patients and providers reporting each response category (count/denominator and percentage). During the patient and provider IDIs, participants were asked to discuss the anticipated effect of regular MedViewer use on patient-provider communication and relationships.

### Statistical Analysis of Quantitative Data

#### General Approach

As this was a feasibility study, our analytic approach focused on calculating precise estimates of the outcomes. Descriptive statistics of categorical variables (eg, race, sex, gender identity, and VL cohort) were presented as counts and percentages, and descriptive statistics of continuous variables (eg, age) were presented as means with SDs or median, IQR, and ranges. We plotted and visually inspected the distribution of continuous variables. Generally, we did not anticipate variable transformations other than those prespecified in the Study Objectives and End Points and Outcome Variables and Measures sections (eg, log_10_ VL).

#### Statistical Analyses

With respect to estimation, a maximum likelihood estimate will be presented together with a 95% CI for the proportion or arithmetic mean of a continuous variable. Estimated proportions will be presented with a corresponding 95% Wilson-Score CI, and estimated means will be presented with a corresponding t-distribution 95% CI. If an estimated proportion is unexpectedly near the 0 or 1 boundary (eg, <0.1 or >0.9), an exact Clopper-Pearson 95% CI will be used as a sensitivity analysis.

Main protocol analyses will be conducted within the 2 enrolled VL strata separately as the strata sample sizes were selected for stratum-specific estimation and precision. We anticipate that patients in group B will be oversampled for study participation compared with the general clinic population. At the time of final analysis, data from the clinic cohort database will be used to define weights for each VL stratum such that a combined, weighted analysis can be used to generalize back to the clinic population. Details of a combined analysis will be prespecified in a separate analysis plan with consideration of additional patient characteristics such as gender, race and ethnicity, and age. Some exploratory analyses may be conducted using the 2 VL groups.

### Qualitative Data Analysis

Data analysis consisted of 4 key steps. The first was reading for content. We began with data reading until the content became intimately familiar. As data were reviewed, emergent themes were noted. The second step was coding. A list of structural codes related to the interview questions was developed. Code definitions were documented in a codebook. Qualitative research assistants were trained to apply the codes using software for qualitative analysis. The codebook was piloted with 5 interview transcripts—each transcript was double coded to reconcile code application, and codes and rules for their application were modified as needed. To ensure intercoder reliability, 100% of the data were double coded. Independent coders reviewed areas of discrepancy until complete agreement was achieved on coded text. The third step was data reduction. We summarized participant responses pertaining to each interview topic and described variation in responses between individuals or among subgroups. We worked with the data related to each code to identify principal subthemes that reflected finer distinctions in the data. The fourth step was data display. Matrices and tables that categorize and display data were used to help facilitate comparisons (eg, across VL strata).

### Primary Sample Size Consideration

We estimated the probability that participants would have their hair sample–based MedViewer report delivered as planned (for primary feasibility) within each VL strata with a corresponding 95% Wilson-Score binomial CI. The same approach was used for acceptability, appropriateness, and additional binary end points. A sample size of 50 would enable sufficient precision for a feasibility study to estimate main and secondary outcomes as well as sufficient power for exploratory data analyses.

## Results

The Establishing Novel Antiretroviral Imaging for Hair to Elucidate Nonadherence study was approved by the local IRB on November 4, 2019. Provider participant enrollment began on January 17, 2020, and patient participant enrollment began on January 22, 2020. Participant enrollment was halted on March 16, 2020, because of the COVID-19 pandemic. When participant enrollment was halted, there were 16 provider participants and 10 patient participants on study. Study activities resumed on February 2, 2021, with COVID-19 modifications approved by the local IRB. Participant enrollment closed on October 8, 2021, and data collection was closed on November 15, 2021. In total, 36 unique patient participants, representing 37 samples, and 20 provider participants were enrolled. Data analysis and manuscript writing will take place into 2023.

## Discussion

### Expected Findings

Over the past 10 years, hair analysis has gained importance in forensic sciences, drug testing [15,16], toxicology investigations [17], and drug adherence [18]. Hair is unique in that it has the potential to provide information about drug intake over a longer period compared with other biological fluids, including plasma [4], blood cells [5], and urine [6]. Advances in bioanalytical technology have transitioned hair analysis from gas chromatography–mass spectrometry methods to more efficient and sensitive liquid chromatography triple-quadrupole mass spectrometry (LC-MS/MS) methods. However, several limitations exist. Some ARVs require collecting a thatch of up to 100 strands of hair [19]. As no one multiplex LC-MS/MS method exists to measure all ARVs in a single sample, multiple thatches are required for complete ARV evaluation. This can be a considerable deterrent for individuals with short hair, those who require hair collected between braids, or those who object to the collection of large amounts of hair for cultural reasons [20]. Although sensitive and specific, LC-MS/MS data require at least 7 steps to process hair for analysis, including segmentation, washing and decontamination, cutting or grinding, extracting, and purifying before analysis can occur [21].

Conversely, our IR-MALDESI MSI method uses single hair strands for analysis. Our method can also determine the identity and distribution of multiple drugs and their metabolites in biological matrices with 1 test without complicated labeling approaches [22-24]. IR-MALDESI MSI requires minimal sample processing, allowing sample analysis to be completed within 2 hours of collection.

Poor medication adherence is widespread among those living with HIV and on lifelong ART [25]. Research, including our statewide survey of North Carolina HIV health care providers, has found that physicians face challenges when counseling their patients about ART adherence [26-34] and that health care professionals would benefit from additional support to effectively address adherence [34-36]. Our research, and that of others, has demonstrated that counseling with accurate adherence feedback can enhance medication adherence [25,37-42].

Therefore, this paper described the rationale and method of a new adherence-enhancing intervention that is based on a novel measure using IR-MALDESI MSI of a hair sample to generate a longitudinal ARV adherence report as objective feedback during a routine clinic visit. This technique is noninvasive and can show daily medication response for the previous month depending on the length of the hair tested. In this study, we aimed to transform these medication responses into easily digestible visual aids for both patients and providers.

Our model is unique in that it assesses both the IR-MALDESI assay and the social behavioral impact of the intervention on the patient’s motivation to adhere to their medication. Furthermore, the study measures the perceived impact on providers’ abilities to counsel their patients and their ability to implement MedViewer in the clinic.

We anticipate that the data collected in this study will provide important insights regarding the feasibility and acceptability of incorporating this new tool into routine clinical care and accentuate potential barriers to medication adherence among patients.

### Limitations

This study allowed participants to self-report data about hair treatments (which could make them ineligible), medication adherence, and social behaviors. Self-report measures in research may present social desirability biases as well as recall bias. On patient and provider questionnaires, respondents may have underreported socially undesirable attitudes or answered questions in a favorable manner. To mitigate social desirability bias, questionnaires were self-administered on computers or tablets whenever possible, and the confidentiality of responses was made clear to participants. On the basis of statistical power calculations, 50 patient participants were anticipated to enroll in this pilot study. This limited sample size may limit our ability to detect differences for exploratory comparisons per strata. Another limitation of this study was the recruitment challenges surrounding patients in group B (detectable VL). Barriers included cancellations, no-shows, lost contact, and a very small number of patients with detectable plasma HIV RNA in the clinic. Hair sampling also has limitations, including needing to exclude patients with bald or shaved heads. A total of 6.7% (10/150) of potentially eligible patients were ineligible because of either having insufficient hair on their head or having recently treated their hair with chemical products. Of the eligible patient participants contacted about taking part in the study, 16% (24/150) declined participation for various reasons, including time constraints, distance lived from clinic, and no longer wanting to participate in research as a whole. Of the eligible providers contacted about study participation, 16% (5/32) declined to take part and 28% (9/32) never responded to contact attempts.

The COVID-19 pandemic presented unique challenges that required a protocol amendment after the first 27% (10/37) of patient participants were enrolled in the study. The remaining 73% (27/37) of patient participants were enrolled using the methods in the protocol amendment. The amendment was designed to limit in-person contact and ensure the safety of patients, research staff, and providers. Revisions to our methodology included redefining a real-time visit as “delivery of the report to the designated research staff member within 2 hours of initiation of hair processing” rather than “...within 2 hours of hair sample collection.” Furthermore, hair collection during study visit 2 (IDIs) was removed from the protocol. Thus, patient adherence over time was not assessed via hair during the pandemic. To increase safety and flexibility, patient visits were conducted in a web-based format, and telehealth visits were available to patients and providers. Web-based data collection may have affected the results. The nature of the pandemic may have also created interpersonal challenges that discouraged patients from being contacted, seeking care, or expressing interest in participating in research.

### Conclusions

To advance the understanding of adherence issues and help inform future research, this project provides insights on the feasibility, acceptability, and appropriateness of integrating MedViewer as an adherence monitoring tool into real-time clinical visits. A strength of this study is that it includes both patient and provider participants to measure the impact on patient behavior and whether it assists providers in their discussions with patients.

This project will contribute to the knowledge gap in the HIV adherence literature and have an effect on ending the HIV and AIDS epidemic. It will advance research by shaping important goals of public health, improving patients’ medication adherence, and facilitating a semistructured method for providers to counsel their patients regarding adherence. Data from this study will determine whether having a broader, more comprehensive picture of how well a patient has adhered to their medication approximately 30 days before a routine clinic visit provides patients and their providers with a motivational tool with detailed information to engage in or sustain medication adherence.

## Appendix

Multimedia Appendix 1 Provider communication aids.

## Abbreviations

ART: antiretroviral therapy
ARV: antiretroviral
CASI: computer-assisted self-interviewing
DTG: dolutegravir
FTC: emtricitabine
HIPAA: Health Insurance Portability and Accountability Act
ID: infectious disease
IDI: in-depth interview
IR: infrared
IRB: institutional review board
LC-MS/MS: liquid chromatography triple-quadrupole mass spectrometry
MALDESI: matrix-assisted laser desorption electrospray ionization
MSI: mass spectrometry imaging
UNC: University of North Carolina at Chapel Hill
VL: viral load

## Data Availability

This study was conducted in accordance with the National Institutes of Health (NIH) public access policy, the NIH data sharing policy, the policy on the dissemination of NIH-funded clinical trial information, and the clinical trials registration and results information submission rule. As such, this trial was registered at ClinicalTrials.gov, with results archived on ClinicalTrials.gov. In addition, results were published in peer-reviewed journals and archived on PubMed Central.
