# Supplementary figures and images for: Establishing Novel Antiretroviral Imaging for Hair to Elucidate Nonadherence: Protocol for a Single-Arm Cross-sectional Study
Source: JMIR Res Protoc. 2023 Apr 21;12:e41188. doi: 10.2196/41188 (PMC10163405; doi:10.2196/41188)

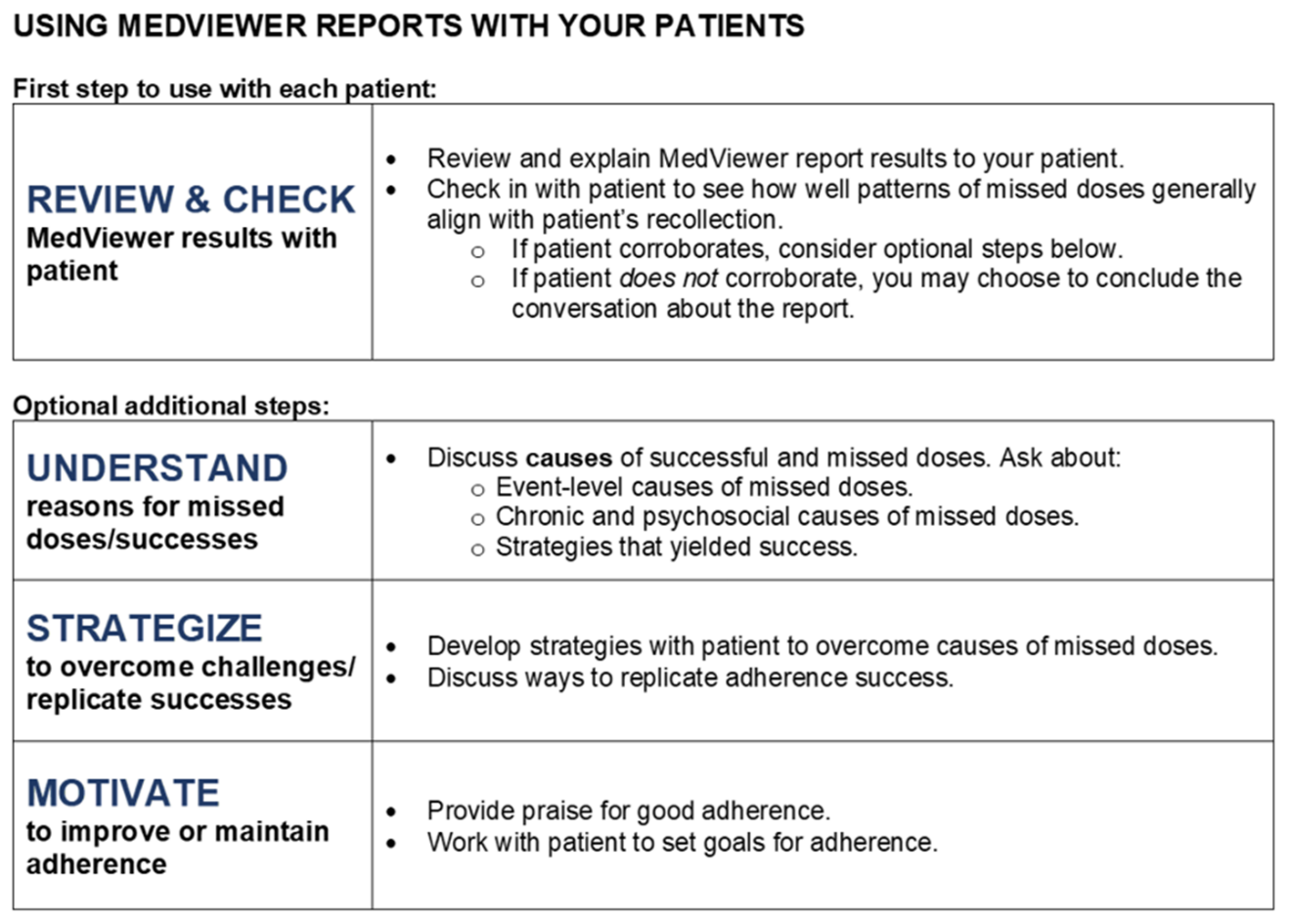

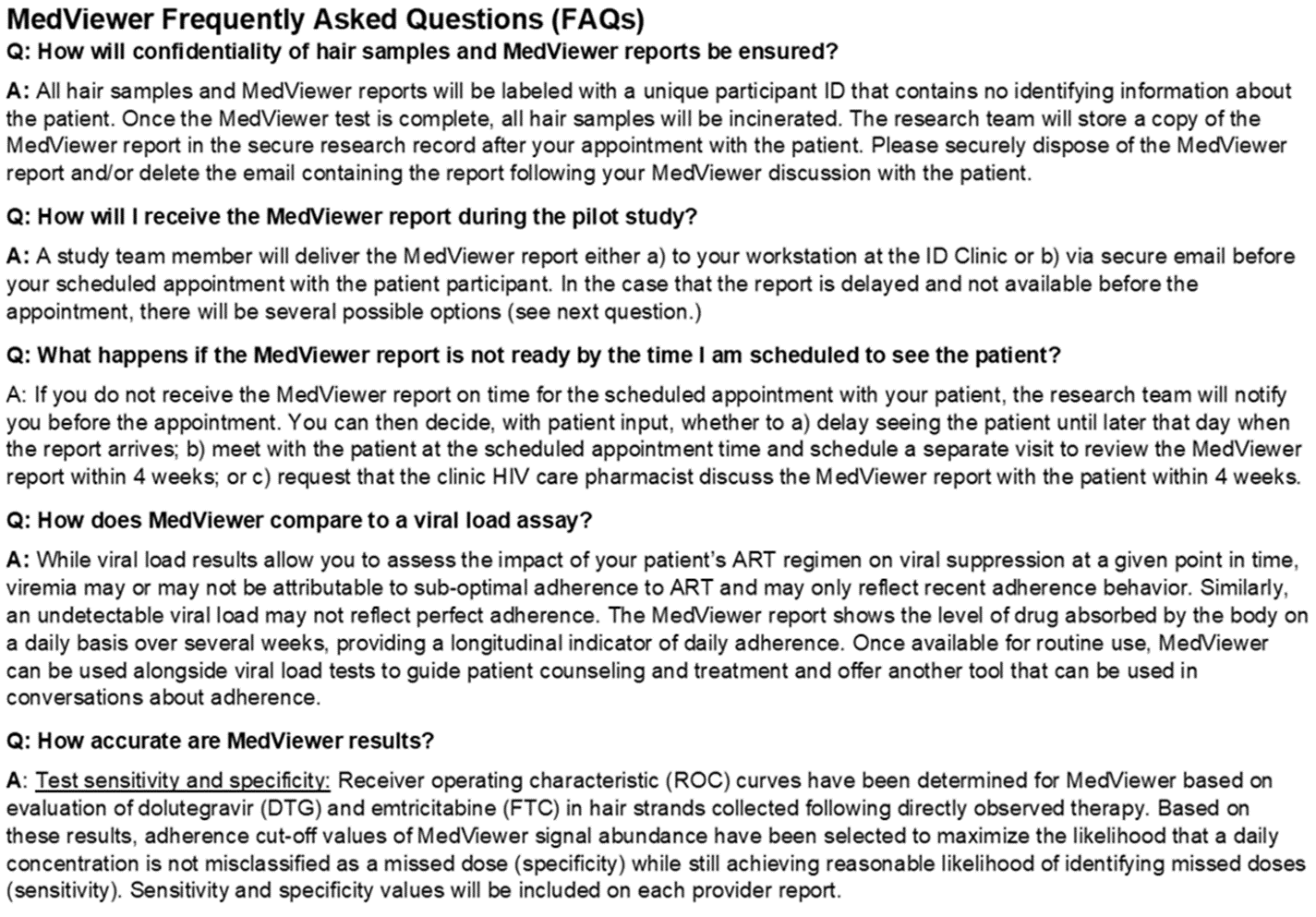

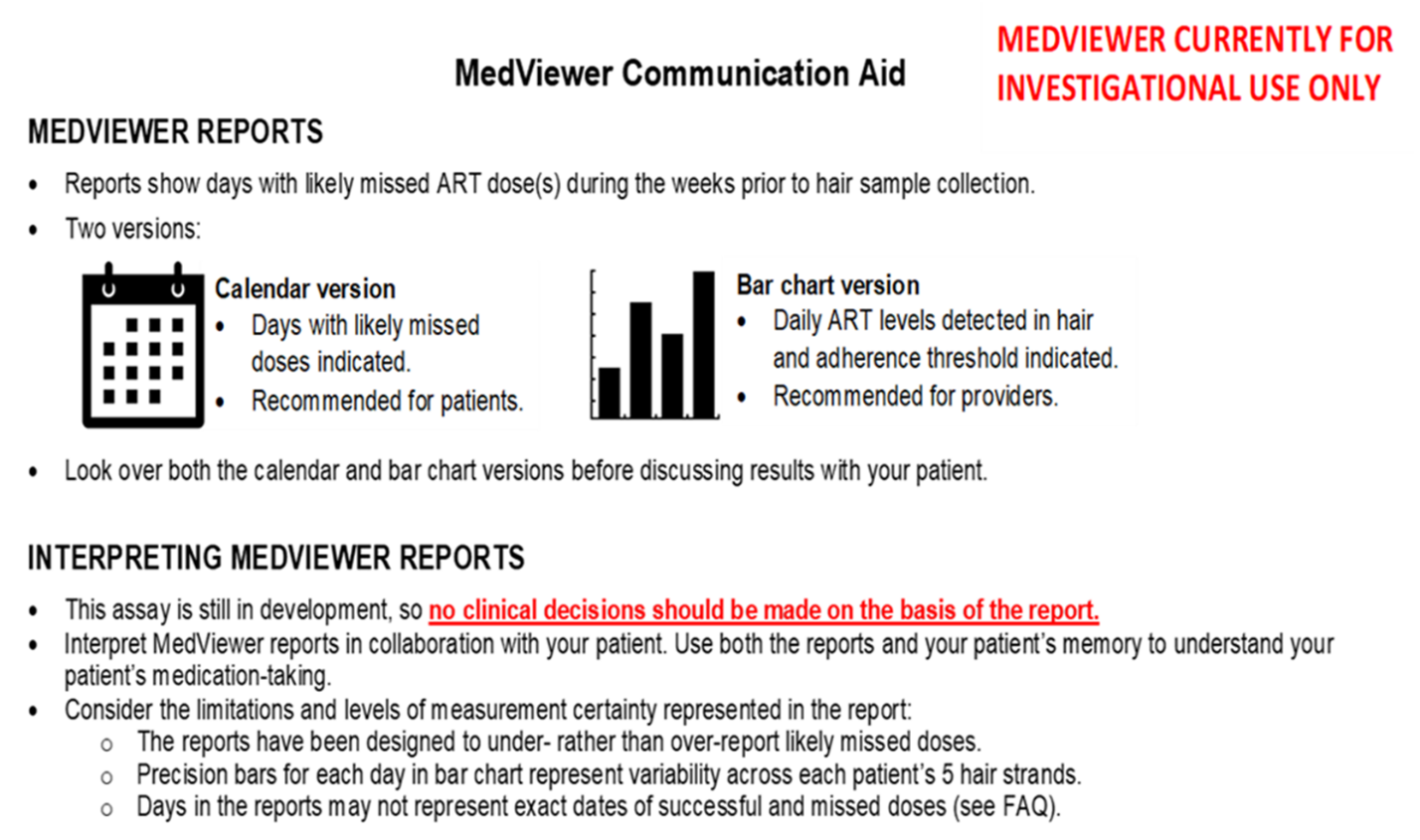

Supplement: Multimedia Appendix 1 [file resprot_v12i1e41188_app1.docx]
